# A phase II trial of dose-dense chemotherapy, followed by surgical resection and/or thoracic radiotherapy, in locally advanced thymoma: report of a Japan Clinical Oncology Group trial (JCOG 9606)

**DOI:** 10.1038/sj.bjc.6605731

**Published:** 2010-06-15

**Authors:** H Kunitoh, T Tamura, T Shibata, K Takeda, N Katakami, K Nakagawa, A Yokoyama, Y Nishiwaki, K Noda, K Watanabe, N Saijo

**Affiliations:** 1 Department of Medical Oncology, National Cancer Center Hospital, 5–1–1 Tsukiji, Chuo-ku, Tokyo 104–0045, Japan; 2 JCOG Data Center, Center for Cancer Control and Information Services, National Cancer Center, 5–1–1 Tsukiji, Chuo-ku, Tokyo 104–0045, Japan; 3 Department of Medical Oncology, Osaka City General Hospital, 2–13–22 Miyakojima-Hondori, Miyakojima-ku, Osaka 534–0021, Japan; 4 Pulmonary Unit, Kobe City Medical Center General Hospital, 4–6 Minatojimanakamachi, Chuo-ku, Kobe, Hyogo 650–0046, Japan; 5 Department of Medical Oncology, Kinki University School of Medicine, 377–2 Ohnohigashi, Osakasayama, Osaka 589–8511, Japan; 6 Department of Medical Oncology, Niigata Cancer Center, 2–15–3, Kawagishi-cho, Niigata-shi, Niigata 951–8566, Japan; 7 Department of Thoracic Oncology, National Cancer Center Hospital East, 6–5–1 Kashiwanoha, Kashiwashi, Chiba 277–8577, Japan; 8 Division of Thoracic Oncology, Kanagawa Cancer Center, 1–1–2 Nakao, Asahi-ku, Yokohama, Kanagawa 241–0815, Japan; 9 Department of Respiratory Medicine, Yokohama Municipal Citizen's Hospital, 56 Okazawa-cho, Hodogaya-ku, Yokohama, Kanagawa 240–0062, Japan; 10 Department of Respiratory Medicine, Mitsui Memorial Hospital, 1 Kandaizumicho, Chiyoda-ku, Tokyo 101–8643, Japan; 11 National Cancer Center Hospital East, 6–5–1 Kashiwanoha, Kashiwashi, Chiba 277–8577, Japan

**Keywords:** chemotherapy, dose-dense, radiotherapy, surgical resection, thymoma, unresectable

## Abstract

**Background:**

This study aimed to evaluate the safety and efficacy of dose-dense weekly chemotherapy, followed by resection and/or thoracic radiotherapy.

**Methods:**

Patients with histologically documented thymoma with unresectable stage III disease received 9 weeks of chemotherapy: cisplatin 25 mg m^−2^ on weeks 1–9; vincristine 1 mg m^−2^ on weeks 1, 2, 4, 6 and 8; and doxorubicin 40 mg m^−2^ and etoposide 80 mg m^−2^ on days 1–3 of weeks 1, 3, 5, 7 and 9. Patients went on to surgery and post-operative radiotherapy of 48 Gy; those with unresectable disease received 60 Gy radiotherapy.

**Results:**

total of 23 patients were entered. The main toxicities of the chemotherapy regimen were neutropenia and anaemia, and 57% of patients completed the planned 9 weeks of therapy. There were no toxic deaths. Of the 21 eligible patients, 13 (62%) achieved a partial response (95% confidence interval: 38–82%). Thirteen patients underwent a thoracotomy and nine (39%) underwent complete resection. Progression-free survival at 2 and 5 years was 80 and 43%, respectively. Overall survival at 5 and 8 years was 85 and 69%, respectively. Survival did not seem to be affected by resection.

**Conclusion:**

In thymoma patients, weekly dose-dense chemotherapy has activity similar to that of conventional regimens. Although some patients could achieve complete resection, the role of surgery remains unclear.

Thymoma is one of the most common tumours to originate in the mediastinum (Giaccone, 2005; Girard *et al*, 2009). Although its clinical behaviour tends to be indolent, it eventually disseminates into the pleural space or sometimes leads to distant metastases. Masaoka's classification has been widely used for clinical staging (Masaoka *et al*, 1981; Girard *et al*, 2009).

The majority of thymomas are discovered at a limited stage, Masaoka's stage I or II, and surgical resection is the treatment of choice for such cases (Giaccone, 2005; Girard *et al*, 2009). Even when the tumour invades neighbouring organs, namely, stage III disease, surgical resection with post-operative radiotherapy is the preferred treatment when the tumour can be completely resected (Curran *et al*, 1988; Urgesi *et al*, 1990; Ogawa *et al*, 2002; Strobel *et al*, 2004).

However, for stage III, unresectable tumours, a combination of chemotherapy and radiotherapy with or without surgical resection is frequently used, but optimal management remains controversial (Ciernik *et al*, 1994; Loehrer *et al*, 1997; Kim *et al*, 2004; Mangi *et al*, 2005; Lucchi *et al*, 2006). There are very few prospective trials with limited numbers of cases, some including stage IV cases (Loehrer *et al*, 1997; Kim *et al*, 2004; Girard *et al*, 2009).

On the other hand, thymomas are generally reported to be chemotherapy-sensitive tumours, with a response rate of 50–70% to combination chemotherapy (Loehrer *et al*, 1994, 1997, 2001; Giaccone *et al*, 1996; Berruti *et al*, 1999; Kim *et al*, 2004; Lucchi *et al*, 2006; Yokoi *et al*, 2007). Active agents include cisplatin (CDDP), vincristine (VCR), doxorubicin (ADM), etoposide (ETP), cyclophosphamide (CPM) and ifosfamide (IFX).

Dose-dense chemotherapy with the CODE combination (CDDP–VCR–ADM–ETP), combined with granulocyte colony-stimulating factor (G-CSF), has been shown to be safe when administered to patients with advanced lung cancer (Murray *et al*, 1991; Fukuoka *et al*, 1997). Theoretically, it might be suitable for chemo-sensitive tumours such as small-cell lung cancers and thymomas, especially in cases with limited tumour burden (Goldie and Coldman, 1983; Levin and Hryniuk, 1987; Murray, 1987). Because of the pilot data in Japan that had suggested that administration of 12 weeks of CODE chemotherapy was barely feasible, subsequent Japanese trials used a modified schedule that was shortened to 9 weeks (Fukuoka *et al*, 1997; Furuse *et al*, 1998).

In 1996, we, the Japan Clinical Oncology Group (JCOG), initiated two clinical trials for advanced thymoma: one aimed to evaluate the safety and efficacy of the CODE regimen in stage IV, disseminated thymoma (JCOG 9605), and the other aimed to evaluate the safety and efficacy of CODE combination chemotherapy, followed by surgical resection and post-operative radiotherapy, in initially unresectable stage III thymoma (JCOG 9606). The primary end point in each study was progression-free survival (PFS). The results of JCOG 9606 are reported herein.

## Patients and methods

### Eligibility criteria

Patients with previously untreated, histologically documented thymomas with Masaoka's stage III disease that was judged to be unresectable by the surgeons, radiologists and medical oncologists at each institute were eligible for entry. Thymoma had to be confirmed histologically, and thymic tumours with other histology, such as thymic carcinoma, carcinoid or lymphoma, were excluded. Each patient was required to fulfil the following criteria: 15–70 years of age; Eastern Cooperative Oncology Group (ECOG) performance status, 0–2; and adequate organ function, that is, leukocyte count ⩾4000/*μ*l, platelet count ⩾10^5^/*μ*l, haemoglobin ⩾10.0 g per 100 ml, serum creatinine <1.5 mg per 100 ml, creatinine clearance ⩾60 ml min^−1^, serum bilirubin <1.5 mg per 100 ml, serum alanine aminotransferase and aspartate aminotransferase less than double the upper limit of the institutional normal range, PaO_2_ ⩾70 mm Hg and predicted post-operative forced expiratory volume in 1 s to be 50% or more of the age-, sex- and height-predicted vital capacity. The exclusion criteria included patients with uncontrolled heart disease, uncontrolled diabetes or hypertension, pulmonary fibrosis or active pneumonitis as evident on chest X-ray, infections necessitating systemic use of antibiotics, disease necessitating emergency radiotherapy, such as superior vena cava obstruction syndrome, active concomitant malignancy, as well as pregnant or lactating women. Also excluded were those with grave complications of thymoma, such as pure red cell aplasia or hypogammaglobulinaemia; myasthenia gravis was allowed and these patients were not excluded *per se*.

Patient eligibility was confirmed by the JCOG Data Center before patient registration. This study protocol was confirmed by the JCOG protocol committee, and then approved by the institutional review boards at each participating centre. Written informed consent was obtained from all patients.

### Treatment plan

#### Chemotherapy

Patients received the 9-week CODE combination chemotherapy described below. Each chemotherapeutic agent was administered intravenously.

Week 1: CDDP 25 mg m^−2^ on day 1 with antiemetics and ample hydration; VCR 1 mg m^−2^ on day 1; ADM 40 mg m^−2^ on day 1; and ETP 80 mg m^−2^ on days 1–3.
Weeks 2, 4, 6 and 8: CDDP 25 mg m^−2^ on day 1 with antiemetics and ample hydration and VCR 1 mg m^−2^ on day 1.
Weeks 3, 5, 7 and 9: CDDP 25 mg m^−2^ on day 1 with antiemetics and ample hydration, ADM 40 mg m^−2^ on day 1 and ETP 80 mg m^−2^ on days 1–3.

Each week, G-CSF (filgrastim 50 *μ*g m^−2^ per day or lenograstim 2 *μ*g kg^−1^ per day) was administered by subcutaneous injection, except on days when chemotherapy was administered or when the leukocyte count was ⩾10 000/*μ*l. Corticosteroid was used only as part of the antiemetic regimen, and the specific drug and dosage were not regulated by the protocol.

Dose and schedule modifications were carried out as previously reported (Kunitoh *et al*, 2009).

### Surgery and radiotherapy

When the tumour was clinically judged to be resectable by the surgeons, radiologists and medical oncologists in each institution, surgical resection of the tumour and a total thymectomy were performed within 6 months (preferably within 3 months) after completion of chemotherapy. For completely resected tumours, post-operative thoracic radiotherapy up to 48 Gy/24 fractions was administered to the surgical margin and the mediastinum. For incompletely resected or unresected tumours, thoracic radiotherapy of up to 60 Gy/30 fractions was administered to the mediastinum and the residual tumour with 1.5-cm margins. The radiation dose per fraction, 2 Gy, and the total doses were determined by the study group in view of previous reports (Girard *et al*, 2009). The actual treatment delivery method was determined at each institution.

Thoracic radiotherapy was started with a linear accelerator (⩾4 MeV) within 6 months of surgery or, for those who did not undergo surgery, on completion of chemotherapy.

#### Patient evaluation and follow-up

Before enrolment into the study, each patient underwent a complete medical history and physical examination (including neurological examination for signs of myasthenia gravis), blood cell count determinations, serum biochemistry testing, arterial blood gas analysis, pulmonary function test, electrocardiogram, chest X-ray, computed tomography (CT) scan of the chest, CT scan or ultrasound of the upper abdomen, whole-brain CT or magnetic resonance imaging and an isotope bone scan. Blood cell counts were determined, serum biochemistry testing was carried out and chest X-rays were taken weekly during each course of chemotherapy.

The toxicity of the chemotherapy was evaluated according to the Japan Clinical Oncology Group Toxicity Criteria (Tobinai *et al*, 1993), modified from the National Cancer Institute Common Toxicity Criteria (NCI-CTC) version 1. Tumour responses were assessed radiographically according to the standard, two-dimensional WHO criteria (Miller *et al*, 1981) and classified into complete response (CR), partial response (PR), no change (NC), progressive disease (PD) and non-evaluable. Response confirmation at 4 weeks or longer intervals was not required in the protocol. After completion of the protocol therapy, the patients were followed up with periodic re-evaluation, including chest CT every 6 months for the first 2 years and yearly thereafter.

#### Central review

Radiographic reviews for eligibility of the enrolled patients and their clinical responses were carried out at the time of the study group meetings. The study coordinator (HK) and a few selected investigators reviewed the radiographic films. The clinical data presented below were all confirmed by this central review. Reviews of pathological specimens were not carried out, because the logistics of the study group were insufficient at the time of study activation in 1997.

### End points and statistical considerations

Because of the rarity of the tumour and the accrual to the US trials (Loehrer *et al*, 1994, 1997), we presumed that we would be capable of accruing 30 patients in the target accrual period of 4 years. The sample size was, therefore, not based on statistical calculations. The expected 5-year PFS rate was 60%, which would give a 95% confidence interval of 40–77% with 30 cases.

Hence, the initial study design envisioned enrolment of 30 fully eligible cases over 4 years, with a follow-up period of 5 years.

The secondary end points included toxicity and safety, objective tumour response to chemotherapy, pattern of relapse, overall survival (OS) and complete resection rate.

The PFS and OS were calculated from the date of enrolment and estimated by the Kaplan–Meier method. Progression-free survival was censored at the date verifiable to be progression free, and OS was censored at the date of last follow-up. During the accrual period, an interim analysis for futility was planned after half of the patients were registered and followed up for at least 6 months. All analyses were performed using SAS software version 8.2/9.1 (SAS Institute, Cary, NC, USA).

## Results

### Patient characteristics

A total of 23 patients from eight institutions were enrolled from July 1997 to April 2005, when the study was terminated because of slow accrual. Two patients were ineligible because of wrong histology; one had thymic carcinoma, and the other had lymphoma. These mistakes occurred because of technical problems in the patient registry. As the ineligible cases did receive the protocol therapy, all 23 patients were analysed for characteristics and toxicity. In all, 21 eligible patients were analysed for clinical response, survival (PFS and OS) and surgical results. The patients’ characteristics are shown in Table 1. Diagnostic procedure was CT-guided needle biopsy in most of the cases.

Reasons for surgical unresectability (one patient could have more than one reason) included invasion into the following: the pulmonary artery trunk in 10 cases, superior vena cava in 8, aorta in 6, extensive pericardium or myocardium in 4, and sternum in 1.

### Chemotherapy delivery and toxicity

Thirteen patients (57%) received the planned 9 weeks of chemotherapy. The other 10 patients included 2 who received 8 weeks, 5 who received 7 weeks, 2 who received 6 weeks and 1 who received 1 week of therapy. Reasons for ceasing chemotherapy were patient refusal (six cases), attending doctors’ decision for earlier local therapy (two cases), disease progression (one case) and ineligibility (one case). The median duration of chemotherapy for the 13 patients who underwent the planned 9 cycles was 9 weeks (range: 9–12 weeks). Among the nine patients who received 6–8 cycles, six received chemotherapy without delay and the remaining three received chemotherapy with a delay of 1–4 weeks.

Table 2 summarises the major toxicities of the chemotherapy. They were mainly haematological, and although about half of the patients experienced grade 4 neutropenia, it was generally transient and complicated by infection in only 1 case. Substantial anaemia was frequently observed, consistent with other reports of dose-dense CODE therapy (Fukuoka *et al*, 1997; Furuse *et al*, 1998). Overall, the toxicities were well tolerated. There were no deaths related to toxicity.

### Clinical response to induction therapy

The clinical responses of the 21 eligible patients to the chemotherapy were judged radiologically and confirmed by central review. The responses were as follows: CR, 0; PR, 13; NC, 7; and PD, 1. The overall response rate was 62% (95% confidence interval: 38–82%).

### Surgical and pathological results

Of the 21 eligible patients, a thoracotomy was performed in 13 (62%). Thoracotomy was performed 26–73 days (median: 47 days) after completion of chemotherapy. The results of the surgery were as follows: probe thoracotomy, two cases; gross residual tumour (R2 resection), one case; microscopically residual tumour on pathological review (R1 resection), one case; and complete surgical and pathological resection (R0 resection), nine cases (43% of all eligible cases). A combined resection of the adjunct organs included pericardium in eight, lung parenchyma in eight, pleura in seven, superior vena cava in two, brachiocephalic vein in two and others in five cases. Pathological CR (pCR), with no residual viable tumour cells in resected specimens, was achieved in three patients (14% of the 21 eligible patients).

The major post-operative morbidities included one case of pulmonary infarction, which subsequently recovered.

### Boost radiotherapy

Post-operative radiotherapy was administered to 7 of the 13 patients who underwent thoracotomy: four of the nine patients with R0 resection received radiotherapy of 48, 48, 48 and 8 Gy, respectively; one of the two patients with incomplete resection received radiotherapy of 50 Gy; and each of the two patients with probe thoracotomy received radiotherapy of 60 Gy. Reasons for not carrying out radiotherapy included surgery-related complication or incomplete recovery (three cases), disease progression (two cases) and patient refusal (one case). Of the eight patients without thoracotomy, five received radiotherapy, with a dose of 60 Gy for each case. The other three patients refused radiotherapy.

**Figure 1:**
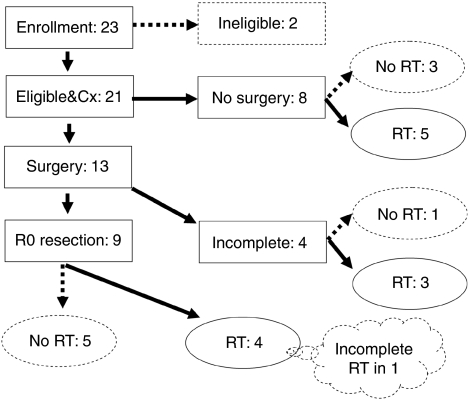
Study schema of the Japan Clinical Oncology Group (JCOG) 9606 trial, with the number of patients who actually received each of the protocol therapies. Cx, chemotherapy; RT, radiotherapy.

The study schema with the actual numbers of patients receiving the protocol therapy is shown in Figure 1.

### Other and late complications

Thirteen patients received thoracic radiotherapy. The toxicities were generally mild and manageable. There were four patients with grade 2 oesophagitis, one patient with a grade 3 skin reaction and another with a grade 2 skin reaction. All other adverse events were grade 0 or 1.

One patient was reported to have pure red cell aplasia, which occurred while receiving post-operative radiotherapy. Radiotherapy was terminated, and the patient recovered with immunosuppressant therapy.

### Progression-free and overall survival

**Figure 2:**
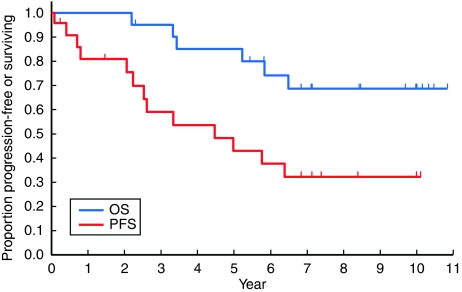
Progression-free survival (PFS) and overall survival (OS) of the 21 eligible patients.

Survival data were last updated in May 2009, 4 years after accrual of the last patient. Figure 2 shows the PFS and OS curves for the 21 eligible cases. The median PFS was 4.5 years (95% confidence interval: 2.3 not calculable years), and the PFS at 2, 5 and 8 years was 80, 43 (95% confidence interval: 21–63%) and 32%, respectively. The median OS was not reached, and the OS at 2, 5 and 8 years was 100, 85 (95% confidence interval: 61–95%) and 69%, respectively.

**Figure 3:**
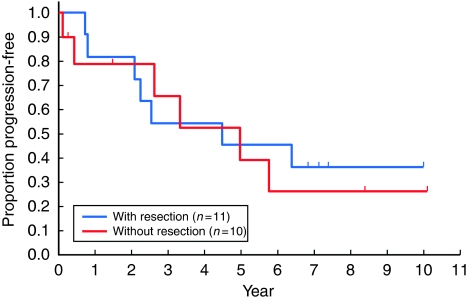
Progression-free survival of the 21 eligible patients, according to the surgery undergone. Resection was performed in 11 patients (complete resection in nine), and 10 patients did not undergo resection (including two with probe thoracotomy). There was no significant difference (log rank *P*=0.75).

**Figure 4:**
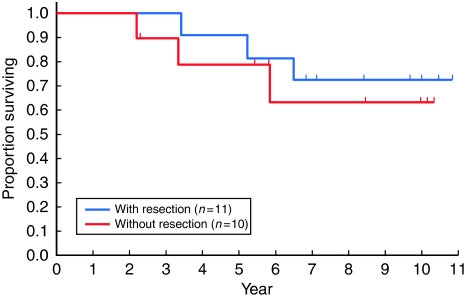
Overall survival of the 21 eligible patients, according to the surgery undergone. Resection was performed in 11 patients (complete resection in nine), and 10 patients did not undergo resection (including two with probe thoracotomy). There was no significant difference (log rank *P*=0.59).

Of the 21 eligible patients, 11 underwent surgical resection (nine complete resection and two incomplete resection), whereas 10 did not (including two who underwent a probe thoracotomy). The PFS and OS were quite similar for those with or without surgical resection. The 5- and 8-year PFS rates for those who underwent resection were 46 and 36% for those with surgical resection and 39 and 26% for those without, respectively (Figure 3). The 5- and 8-year OS rates were 91 and 73% for those with surgical resection and 79 and 63% for those without, respectively (Figure 4).

For the nine patients who underwent R0 resection, the outcomes were marginally better, with 5- and 8-year PFS rates of 56 and 44%, respectively, and 5- and 8-year OS rates of 89 and 78%, respectively. The case with R1 resection had relapse at 2.3 years, and the case with R2 resection had relapse at 0.7 year.

All three patients who achieved pCR were alive and disease free at 6.3–7.4 years of follow-up.

### Pattern of relapse

So far, 13 of the 21 eligible patients have had tumour relapse. All of the 13 relapsed patients initially demonstrated regrowth of the primary and/or pleural or pericardial dissemination: primary only in five; pleura or pericardium only in six, and both in two2. None had initial relapse involving distant organs. There was no report of needle biopsy-track recurrence.

## Discussion

The optimal management of unresectable stage III thymoma remains unclear. There are some reports of combined modality approaches including chemotherapy and surgery, but many reports included stage IV disease and/or thymic carcinoma histology (Berruti *et al*, 1999; Kim *et al*, 2004; Lucchi *et al*, 2006; Yokoi *et al*, 2007). Reports of multicentre prospective trials are very few (Table 3).

In the current trial, we prospectively accrued patients with unresectable stage III thymoma, and excluded thymic carcinoma; it is now evident that thymoma and thymic carcinoma differ in clinical presentation and in prognosis, and trials on them should be reported separately (Eng *et al*, 2004; Giaccone, 2005).

We previously reported the results of another trial, JCOG 9605 (Kunitoh *et al*, 2009), in which we treated patients with stage IV thymoma with CODE chemotherapy. The results were similar to conventional chemotherapy, and we concluded that intensive chemotherapy does not seem to be promising enough in disseminated thymoma. However, dose-dense chemotherapy might still have a role in patients with limited tumour burden, in combination with definitive local therapy.

Although our results showed that CODE chemotherapy in combination with local therapy could be safely administered to thymoma patients, the efficacy was not remarkable. Compliance to chemotherapy was poorer; only 57% of patients completed the planned 9-week schedule, as compared with the 87% rate in the JCOG 9605 study for stage IV disease (Kunitoh *et al*, 2009). Doctors’ and patients’ decisions were the main reasons for ceasing chemotherapy and early local therapy. Therefore, although chemotherapy itself was well tolerated, toxicities such as malaise or fatigue, which the old JCOG toxicity criteria did not define, might have compromised the completion of chemotherapy before surgery.

Moreover, although the sample size was smaller than expected because of poor accrual, the 5-year PFS rate was 43% (95% confidence interval: 21–63%), which fell short of the expected 60%. Although the OS rate was favourable, it would be difficult to make a valid conclusion because of the small sample size (Table 3).

In this study, we did show that about half of the patients with an initially unresectable thymoma were able to undergo complete resection after induction CODE chemotherapy. However, both PFS and OS were surprisingly similar for patients with and without complete resection.

Those who underwent complete resection got numerically better PFS and OS rates, but the difference with unresected cases was marginal, especially considering the selection bias. Only those who received pathological CR enjoyed clearly favourable outcomes. Low compliance to radiotherapy in patients with surgery could partly account for the unexpected results.

Complete resection has been reported to be associated with good prognosis in patients with stage III thymoma (Regnard *et al*, 1996; Girard *et al*, 2009). On the other hand, the role of ‘debulking’ surgery, in patients in whom complete resection is not feasible, remains unclear. Although some have suggested it to be beneficial (Liu *et al*, 2006), others reported that it could not affect the outcome as compared with biopsy only, followed by radiotherapy (Ciernik *et al*, 1994).

Taken together with our results, we believe that the role of surgery in locally advanced thymoma, as compared with definitive radiotherapy, still remains to be established, especially in combination with systemic chemotherapy. More studies are warranted.

One major limitation of the study is that we did not perform a central review of the histology, and thus could not provide WHO classifications of histology (Okumura *et al*, 2002; Travis *et al*, 2004). This makes comparisons with results from other reports difficult. Central pathology review and, preferably, tissue collection would be very important in future trials.

Now JCOG is discussing our next study on thymoma. As intensification of the current chemotherapy does not seem to be promising enough, our next approach would be trials with new agents, cytotoxic (such as amrubicin or irinotecan) or target based. More translational research of the tumour would be necessary, as well as international cooperation, given the rarity of the disease.

In conclusion, we found that weekly dose-dense chemotherapy could be administered safely to patients with thymoma, even when combined with local therapy in localised disease. However, the efficacy seemed to be no better than that of conventional chemotherapy. More research on the optimal systemic therapy, as well as on the role of surgery in locally advanced disease, seems to be necessary.

## Figures and Tables

**Table 1:** Patients’ characteristics

**Item**	**Number**
Sex (male/female)	17/6
Age, years (median/range)	56 (28–70)
	
*ECOG performance status*	
PS0/PS1/PS2	9/14/0
	
*Smoking history*	
No	13
Yes (median pack-years)	10 (28)
Myasthenia gravis (no/yes)	21/2
Histology: thymoma and eligible	21
Lymphocyte predominance	10
Mixed cell	4
Epithelioid cell	6
Spindle cell	1
Histology: not thymoma (ineligible)	2
Carcinoma	1
Lymphoma	1

Abbreviations: ECOG=Eastern Cooperative Oncology Group; PS=performance status.

**Table 2:** Toxicity of the chemotherapy (*N*=23)

**Toxicity**	**Grades 1/2**	**Grade 3**	**Grade 4**	**%Grade 3/4**
Leukopenia	4/5	8	5	57
Neutropenia	1/6	3	11	61
Anaemia	0/3	19	ND	83
Thrombocytopenia	6/4	4	2	26
ALT	10/1	1	0	4
Creatinine	2/0	0	0	0
PaO_2_	5/6	0	0	0
Emesis	10/8	3	ND	13
Diarrhea	3/3	1	0	4
Stomatitis	5/2	0	0	0
Constipation	2/1	0	0	0
Neuropathy	7/2	0	ND	0
Infection	5/2	3	0	13

Abbreviations: ALT=alanine aminotransaminase; ND=not defined (the Japan Clinical Oncology Group toxicity criteria did not define grade 4 in these toxicities).

**Table 3:** Reports of prospective trials of combined modality therapy for locally advanced thymoma

**Treatment**	**Stage**	**Patients^a^**	**ORR**	**5-yr OS**
PAC, R (Loehrer *et al*, 1997)	III	23	70%	52.5%^b^
PAC, S, R (Kim *et al*, 2004)	III/IV	22	77%	95%^c^
CODE, S, R (current study)	III	21	62%	85%^d^

Abbreviations: CODE=combination chemotherapy with cisplatin/vincristine/doxorubicin/etoposide; ORR: overall response rate; PAC=combination chemotherapy with cisplatin/doxorubicin/cyclophosphamide; R=thoracic radiotherapy; S=surgical resection; 5-yr OS=overall survival rate at 5 years.

a Number of assessable patients.

b Including patients with thymic carcinoma.

c 7-year OS rate was 79%.

d 8-year OS rate was 69%.

## Appendix 1

### Study participants

The following institutions and investigators participated in the trial:

National Cancer Center Hospital East (Yutaka Nishiwaki, Kaoru Kubota, Nagahiro Saijo), National Cancer Center Hospital (Tomohide Tamura, Noboru Yamamoto, Hideo Kunitoh), Kanagawa Cancer Center (Kazumasa Noda, Fumihiro Oshita), Yokohama Municipal Citizen's Hospital (Koshiro Watanabe, Hiroaki Okamoto), Niigata Cancer Center Hospital (Akira Yokoyama, Yuko Tsukada), Kinki University Hospital (Kazuhiko Nakagawa, Isamu Okamoto), Osaka City General Hospital (Koji Takeda, Haruko Daga), and Kobe City Medical Center General Hospital (Nobuyuki Katakami, Hisashi Nishimura).
